# Etiology of the Broad Avoidant Restrictive Food Intake Disorder Phenotype in Swedish Twins Aged 6 to 12 Years

**DOI:** 10.1001/jamapsychiatry.2022.4612

**Published:** 2023-02-01

**Authors:** Lisa Dinkler, Marie-Louis Wronski, Paul Lichtenstein, Sebastian Lundström, Henrik Larsson, Nadia Micali, Mark J. Taylor, Cynthia M. Bulik

**Affiliations:** 1 Department of Medical Epidemiology and Biostatistics, Karolinska Institutet, Stockholm, Sweden; 2 Translational Developmental Neuroscience Section, Division of Psychological and Social Medicine and Developmental Neurosciences, Faculty of Medicine, TU Dresden, Dresden, Germany; 3 Neuroendocrine Unit, Department of Medicine, Massachusetts General Hospital, Harvard Medical School, Boston; 4 Gillberg Neuropsychiatry Centre, Institute of Neuroscience and Physiology, Sahlgrenska Academy, University of Gothenburg, Gothenburg, Sweden; 5 School of Medical Sciences, Örebro University, Örebro, Sweden; 6 Mental Health Services in the Capital Region of Denmark, Eating Disorders Research Unit, Psychiatric Centre Ballerup, Copenhagen, Denmark; 7 Great Ormond Street Institute of Child Health, University College London, London, United Kingdom; 8 Department of Psychiatry, University of North Carolina at Chapel Hill; 9 Department of Nutrition, University of North Carolina at Chapel Hill

## Abstract

**Importance:**

Avoidant restrictive food intake disorder (ARFID) is characterized by an extremely limited range and/or amount of food eaten, resulting in the persistent failure to meet nutritional and/or energy needs. Its etiology is poorly understood, and knowledge of genetic and environmental contributions to ARFID is needed to guide future research.

**Objective:**

To estimate the extent to which genetic and environmental factors contribute to the liability to the broad ARFID phenotype.

**Design, Setting, and Participants:**

This nationwide Swedish twin study includes 16 951 twin pairs born between 1992 and 2010 whose parents participated in the Child and Adolescent Twin Study in Sweden (CATSS) at twin age 9 or 12 years. CATSS was linked to the National Patient Register (NPR) and the Prescribed Drug Register (PDR). Data were collected from July 2004 to April 2020, and data were analyzed from October 2021 to October 2022.

**Main Outcomes and Measures:**

From CATSS, NPR, and PDR, all parent reports, diagnoses, procedures, and prescribed drugs that were relevant to the *DSM*-*5* ARFID criteria were extracted when twin pairs were aged 6 to 12 years and integrated into a composite measure for the ARFID phenotype (ie, avoidant/restrictive eating with clinically significant impact, such as low weight or nutritional deficiency, and with fear of weight gain as an exclusion). In sensitivity analyses, autism and medical conditions that could account for the eating disturbance were controlled for. Univariate liability threshold models were fitted to estimate the relative contribution of genetic and environmental variation to the liability to the ARFID phenotype.

**Results:**

Of 33 902 included children, 17 151 (50.6%) were male. A total of 682 children (2.0%) with the ARFID phenotype were identified. The heritability of ARFID was 0.79 (95% CI, 0.70-0.85), with significant contributions from nonshared environmental factors (0.21; 95% CI, 0.15-0.30). Heritability was very similar when excluding children with autism (0.77; 95% CI, 0.67-0.84) or medical illnesses that could account for the eating disturbance (0.79; 95% CI, 0.70-0.86).

**Conclusions and Relevance:**

Prevalence and sex distribution of the broad ARFID phenotype were similar to previous studies, supporting the use of existing epidemiological data to identify children with ARFID. This study of the estimated genetic and environmental etiology of ARFID suggests that ARFID is highly heritable, encouraging future twin and molecular genetic studies.

## Key Points

**Question:**

To what extent do genetic and environmental factors contribute to the liability to avoidant restrictive food intake disorder (ARFID)?

**Findings:**

In this nationwide Swedish twin study including 16 951 twin pairs aged 6 to 12 years, using parent-reported and national health register data, a composite score to identify the ARFID phenotype was developed. The heritability of ARFID was high (79%), and nonshared environmental factors played a smaller but significant role (21%).

**Meaning:**

In this study, the heritability of ARFID was comparable with the heritability of other eating disorders and similar to heritability of neurodevelopmental disorders.

## Teaser

This nationwide twin study evaluates the extent to which genetic and environmental factors contribute to the liability to the broad avoidant restrictive food intake disorder phenotype.

## Introduction

Avoidant restrictive food intake disorder (ARFID) is a serious feeding and eating disorder formally recognized in *DSM*-*5*^1^ in 2013 and first included into the *International Classification of Diseases, 11th Revision* (*ICD*-*11*) in 2022.^2^ Characterized by an extremely limited range or amount of food consumed and resulting in persistent failure to meet nutritional and/or energy needs, ARFID is associated with considerable individual, family, and social impairment,^3^ and medical consequences^4,5^ can be life threatening. Unlike anorexia nervosa, dietary restriction in ARFID is not motivated by body image concerns or drive for thinness but rather based on sensory sensitivity to food qualities (eg, texture, smell, taste), lack of interest in food/eating (ie, low appetite), and/or fear of aversive somatic consequences of food intake (eg, choking, vomiting, allergic reactions),^1^ often in response to aversive eating experiences.^6^ With an estimated prevalence of 1% to 5%,^7,8^ ARFID is at least as common as autism^9^ and potentially as common as attention-deficit/hyperactivity disorder (ADHD).^10^

The etiology of ARFID remains poorly understood, and the genetics of ARFID are understudied. Other eating disorders, such as anorexia nervosa and bulimia nervosa, have been shown to have moderate to high heritability,^11^ and large-scale genome-wide association studies have successfully identified risk loci for anorexia nervosa and underscored the importance of considering metabolic factors in its etiology.^12^ In contrast, the heritability of ARFID is as yet unknown, although twin studies on related phenotypes showed low to moderate heritability of macronutrient, micronutrient, and overall caloric intake (range, 0.21-0.48)^13^ and fruit/vegetable liking (range, 0.37-0.54)^14,15^; and moderate to high heritability of food fussiness (range, 0.46-0.78),^14,16^ food neophobia (range, 0.58-0.78),^16,17,18^ and appetite (range, 0.53-0.84).^19,20^ In addition, heritability of being at high risk of ARFID was significant when estimated from common genetic variants in autistic individuals.^21^

Importantly, the genetic epidemiology of ARFID is unknown because validated ARFID screening instruments are only starting to emerge. Until such measures have been developed and deployed, we can optimize available resources such as those held by the Swedish Twin Registry to create a diagnostic algorithm to identify an ARFID phenotype and study its prevalence, correlates, and etiology. The aim of this study was to determine the extent to which genetic and environmental factors contribute to the liability to ARFID. Based on the moderate to high heritability of other eating disorders (anorexia nervosa: range, 0.48-0.74; bulimia nervosa: range, 0.55-0.61; binge-eating disorder: range, 0.39-0.45)^22^ and the reported heritability estimates of ARFID-related traits, we expected at least moderate heritability of ARFID.

## Methods

### Participants

We leveraged existing data from the Child and Adolescent Twin Study in Sweden (CATSS), targeting all twins born in Sweden since July 1, 1992.^23^ CATSS is one of the largest twin studies in the world, contains a broad range of psychiatric and neurodevelopmental phenotypes, and is linked to national population health and quality registers.^23^ Parents of twins are first invited to participate in CATSS at twin age 9 years (the cohorts born July 1992 to June 1995 were assessed at age 12 years). Zygosity of same-sex twins was ascertained via an extensively validated panel of 47 common genetic variants for 79% of monozygotic twins and 58% of dizygotic same-sex twins.^24^ For the remaining twin pairs, a validated algorithm of 5 questions regarding twin similarity was used.^25^ Only twins with more than 95% probability of being correctly classified were assigned zygosity by this method. Detailed information on race and ethnicity was not available. This study was approved by the Regional Ethical Review Board in Stockholm, Sweden. Informed consent (written and/or oral) was obtained from the parents.

This study included twins born between 1992 and 2010 who were part of CATSS at age 9 or 12 years (response rate approximately 69%). For this sample, data from the National Patient Register (NPR; diagnostic and procedure codes from inpatient care with full coverage since 1987 and approximately 80% of specialized outpatient care since 2001^26^; *ICD*-*9* codes used between 1987 and 1996 and *ICD*-*10* codes used since 1997) were available until the end of 2016. Data from the Prescribed Drug Register (PDR; all dispensations of prescribed drugs since 2005, active drug ingredients coded according to the Anatomical Therapeutic Chemical [ATC] Classification System) were available until the end of 2017.^27^ We excluded twins with unknown zygosity (n = 435) and missing co-twin (n = 45). The final sample included 33 902 individuals (5184 monozygotic pairs, 5936 dizygotic same-sex pairs, and 5831 dizygotic opposite-sex pairs).

### Identification of the ARFID Phenotype

To identify children with the ARFID phenotype, we extracted all information relevant to the *DSM*-*5* criteria for ARFID from CATSS, NPR, and PDR and developed a composite measure (Figure). eTable 1 in Supplement 1 provides a full list of CATSS items, NPR diagnostic and procedure codes, and PDR ATC codes used to evaluate the *DSM*-*5* ARFID criteria.

**Figure.**
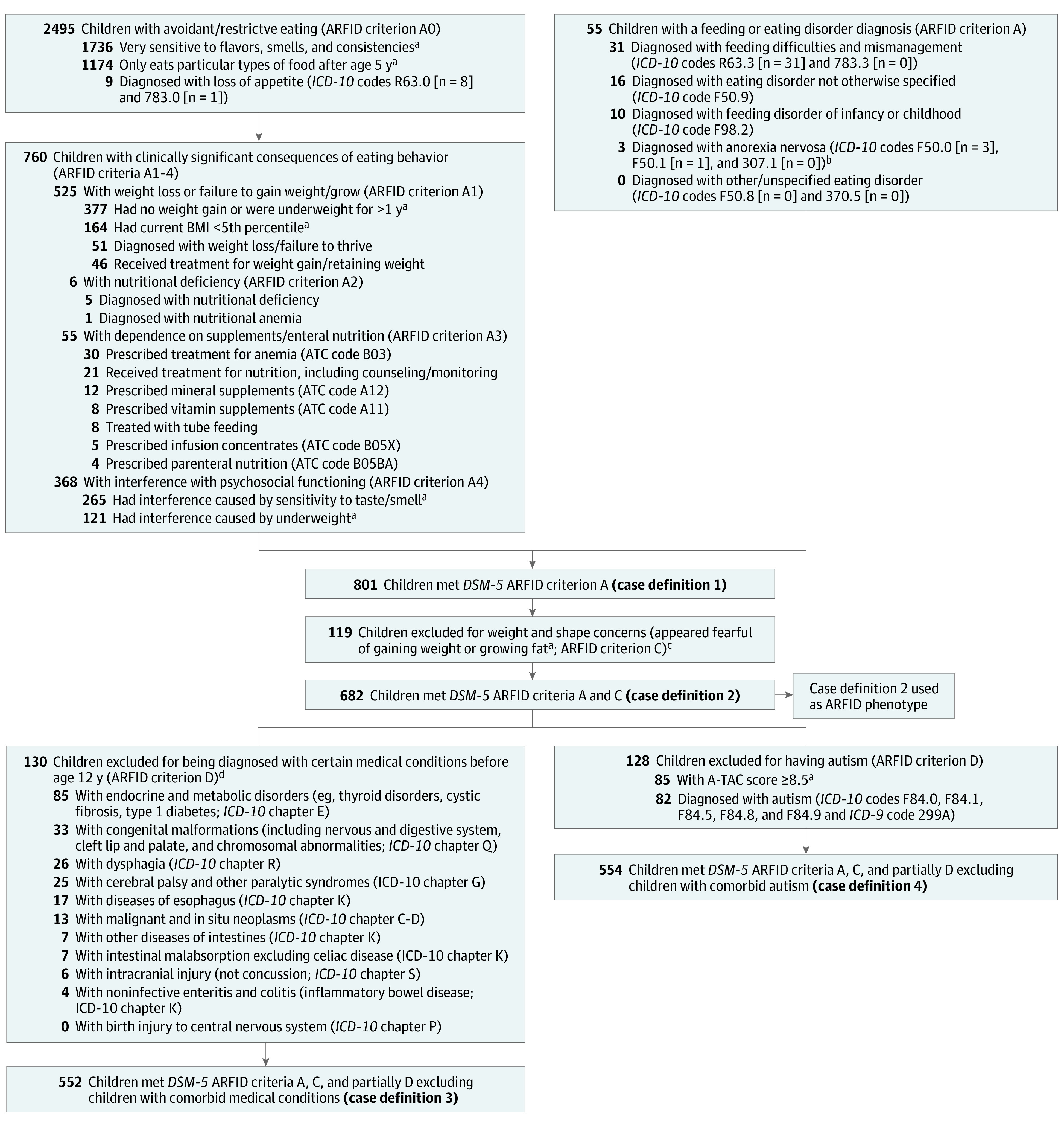
Identification of the Avoidant Restrictive Food Intake Disorder (ARFID) Phenotype Between Age 6 and 12 Years Among 33 902 Twins Diagnoses, procedures, and prescribed drugs between age 6 and 12 years were included. A-TAC indicates Autism-Tics, AD/HD, and Other Comorbidities Inventory; ATC, Anatomical Therapeutic Chemical Classification System; BMI, body mass index; *ICD*-*10*, *International Statistical Classification of Diseases and Related Health Problems, Tenth Revision*. ^a^Variables are parent reported at age 9 or 12 years. ^b^Three individuals received a diagnosis of anorexia nervosa (*ICD*-*10* codes F50.0 or F50.1; 1 individual received both codes) between age 6 and 12 years. Age at first diagnosis was around 11 years and 8 to 9 months for all 3 individuals. One individual was excluded in the next step (ie, did not meet *DSM*-*5* ARFID criteria A and C) because of parent-reported fear of weight gain. ^c^Ten individuals who met ARFID criterion A were removed because of missing response on this item. ^d^Diagnostic codes for medical conditions that could potentially exclude an ARFID diagnosis were selected based on their presence in the sample; therefore, not all medical conditions that could potentially be an exclusion criterion for ARFID are listed here.

#### *DSM*-*5 *ARFID Criterion A

All information from CATSS used in this study was reported by parents, either at twin age 9 years (27 492 of 33 902 children [81.1%]) or at twin age 12 years (6410 of 33 902 children [18.9%]). To match this age range, we included diagnostic and procedure codes from the NPR and prescribed drugs from the PDR to assess *DSM*-*5 *ARFID criterion A (avoidant/restrictive eating with clinically significant consequences of the eating behavior, eg, low weight/failure to thrive, nutritional deficiency, dependence on nutritional supplements, or psychosocial impairment) between age 6 and 12 years. We chose age 6 years to increase sensitivity for potential consequences of the eating disturbance diagnosed earlier than age 9 years, which is the lower age bound for the parent reports from CATSS.

#### *DSM*-*5* ARFID Criterion B

Criterion B (eating disturbance is not better explained by lack of available food or an associated culturally sanctioned practice) could not be considered, as such information was not available; however, the clinical feeding and eating disorder diagnoses and the specific CATSS items used to identify the ARFID phenotype are unlikely to reflect lack of available food or cultural practices causing the eating disturbance (eTable 1 in Supplement 1).

#### *DSM*-*5* ARFID Criterion C

Criterion C (eating disturbance not attributable to anorexia nervosa, bulimia nervosa, or body image disturbance) was evaluated using parent-reported fear of weight gain at age 9 or 12 years.

#### *DSM*-*5* ARFID Criterion D 

To assess criterion D (eating disturbance not attributable to a concurrent medical condition or another mental disorder), we selected a range of medical conditions at any time before age 12 years that could potentially explain the eating disturbance. Furthermore, we wanted to ascertain that genetic and environmental influences on the ARFID phenotype are not only due to autism, which is highly heritable^28^ and often cooccurs with ARFID.^29,30^ Therefore, we identified children with an NPR diagnosis of autism at any point in their life (*ICD*-*9* code 299A; *ICD*-*10* codes F84.0, F84.1, F84.5, F84.8, and F84.9) as well as children scoring above the cutoff (8.5 or more points) on the autism scale of the Autism-Tics, AD/HD, and Other Comorbidities Inventory (A-TAC), which has been well-validated for autism.^31,32,33^

#### Case Definitions

In summary, we identified 4 different case definitions (Figure): (1) children who met *DSM*-*5* ARFID criterion A, (2) children who met *DSM*-*5* ARFID criteria A and C, (3) children who met *DSM*-*5* ARFID criteria A, C, and partially D excluding children with comorbid medical conditions that could potentially explain the eating disturbance, and (4) children who met both *DSM*-*5 *ARFID criteria A, C, and partially D excluding children with comorbid autism. However, medical conditions and autism are common comorbidities of ARFID,^29,30,34^ and in this epidemiological context, it is impossible to determine whether, in each specific case, the selected medical conditions are cause, comorbidity, or consequence of ARFID. Hence, we deemed case definitions 3 and 4 too conversative definitions of ARFID, whereas the case definition 1 was too broad, as it did not exclude children with fear of weight gain. We therefore considered case definition 2 (criteria A and C) to best reflect children with the ARFID phenotype and conducted sensitivity analyses for the other 3 case definitions.

### Statistical Analysis

The twin design is based on comparing the relative similarity of monozygotic and dizygotic twins on a trait, capitalizing on the fact that monozygotic twins are genetically identical whereas dizygotic twins share, on average, 50% of their segregating DNA. In contrast to nontwin siblings, twins are also matched for shared environmental influences by sharing the intrauterine environment and growing up in the same family at the same time. By comparing twin correlations, we can therefore estimate 3 variance components to the phenotype: additive genetics (A), shared environment (C) or dominant genetics (D), and nonshared environment (E) (albeit components C and D cannot be estimated simultaneously, as they confound each other in the classic twin design).

Here, we fitted univariate liability threshold models (which are based on dichotomous data but assume an underlying continuous distribution of liability to the categorical construct) to estimate the relative contribution of genetic and environmental variation to the liability to the ARFID phenotype for each of the 4 case definitions. As little is known about sex differences in ARFID (including sex differences in its clinical presentation, epidemiology, and etiology), we initially fitted a saturated model including quantitative and qualitative sex limitation to the observed data for all 4 case definitions (quantitative sex limitation: genetic and environmental variation influences phenotypic variance to differing degrees in female and male children; qualitative sex limitation: different genetic and environmental influences in female and male children).

Assumption testing for this saturated model revealed no violations of the assumed equal thresholds across twin order and across zygosity in same-sex twin pairs (Table 1; eTable 2 in Supplement 1). Twin correlations were estimated from a constrained saturated model in which the thresholds were equated across twin order and across zygosity within sex (ie, 2 thresholds were estimated, one for all female twins and one for all male twins). All dizygotic same-sex twin correlations were less than half of the monozygotic twin correlations, indicating either dominant genetics or sibling contrast effects (ie, parental emphasis on within-pair differences; Table 2). Twin correlations of monozygotic male twins were somewhat higher than twin correlations of monozygotic female twins, while twin correlations of dizygotic same-sex male twins were somewhat lower than twin correlations of dizygotic female twins, suggesting quantitative sex differences. Qualitative sex differences were only indicated for case definition 3, where the twin correlation of dizygotic opposite-sex pairs was lower than the average of the twin correlations of dizygotic same-sex female twins and dizygotic same-sex male twins. Qualitative sex differences and sibling contrast effects cannot be estimated in the same model, as the model would be underidentified. Since there was little indication of qualitative sex differences, we fitted ADE-s models with only quantitative sex limitation. Sibling contrast effects were modeled by adding a pathway (-s) between one twin’s phenotype and their cotwin’s phenotype. Significance of individual parameters was tested by constraining them to be equal to zero (significance level *P* < .05). The best-fitting models were chosen based on the likelihood ratio test (the reduced model was favored if model fit did not deteriorate significantly). All *P* values were 2-tailed. Data management was performed using SAS version 9.4 (SAS Institute). Data analysis was performed using OpenMx version 2.20.6^35^ (The OpenMx Project) in R version 4.2.0 (The R Foundation).

**Table 1.** Number of Children With the Avoidant Restrictive Food Intake Disorder (ARFID) Phenotype and Proportion of Children Meeting Subcriteria of *DSM*-*5* ARFID Criterion A by Sex, Zygosity, and Case Definition

Characteristic	Case definition, No./total No. (%)^a^			
	1	2^b^	3	4
Total sample	801/33 902 (2.4)	682/33 902 (2.0)	552/33 902 (1.6)	554/33 902 (1.6)
Sex				
Female	342/16 751 (2.0)	267/16 751 (1.6)	210/16 751 (1.3)	229/16 751 (1.4)
Male	459/17 151 (2.7)	415/17 151 (2.4)	342/17 151 (2.0)	325/17 151 (2.0)
Sex and zygosity				
Monozygotic				
Female	99/5384 (1.8)	80/5384 (1.5)	55/5384 (1.0)	68/5384 (1.3)
Male	115/4984 (2.3)	97/4984 (1.9)	78/4984 (1.6)	75/4984 (1.5)
Dizygotic same sex				
Female	127/5536 (2.3)	106/5536 (1.9)	90/5536 (1.6)	92/5536 (1.7)
Male	167/6336 (2.6)	153/6336 (2.4)	127/6336 (2.0)	117/6336 (1.8)
Dizygotic opposite sex				
Female	116/5831 (2.0)	81/5831 (1.4)	65/5831 (1.1)	69/5831 (1.2)
Male	177/5831 (3.0)	165/5831 (2.8)	137/5831 (2.3)	133/5831 (2.3)
Children meeting subcriteria of *DSM*-*5* ARFID criterion A				
A1 (weight loss or failure to gain weight/grow)	545/801 (68.0)	458/682 (67.2)	363/552 (65.8)	358/554 (64.6)
A2 (nutritional deficiency)	6/801 (0.7)	4/682 (0.6)	2/552 (0.4)	2/554 (0.4)
A3 (dependence on supplements/enteral nutrition)	62/801 (7.7)	58/682 (8.5)	24/552 (4.3)	37/554 (6.7)
A4 (interference with psychosocial functioning)	375/801 (46.8)	345/682 (50.6)	296/552 (53.6)	283/554 (51.1)

^a^ Case definition 1 included children who met *DSM*-*5* ARFID criterion A (avoidant/restrictive eating with clinically significant consequences of the eating behavior); case definition 2, children who met *DSM*-*5* ARFID criteria A and C (eating disturbance not attributable to anorexia nervosa, bulimia nervosa, or body image disturbance); case definition 3, children who met *DSM*-*5* ARFID criteria A, C, and partially D (eating disturbance not attributable to a concurrent medical condition or another mental disorder) excluding children with comorbid medical conditions; and case definition 4, children who met *DSM*-*5* ARFID criteria A, C, and partially D excluding children with comorbid autism.

^b^ Case definition 2 best reflects children with the ARFID phenotype.

**Table 2.** Tetrachoric Twin Correlations and Probandwise Concordance Rates for the Avoidant Restrictive Food Intake Disorder (ARFID) Phenotype by Zygosity, Sex, and Case Definition^a^

Case definition	*r* (95% CI)				
	Female		Male		Dizygotic opposite sex
	Monozygotic	Dizygotic same sex	Monozygotic	Dizygotic same sex	
Twin pairs, No.	2692	2768	2492	3168	5831
Twin correlations					
1	0.64 (0.48 to 0.76)	0.31 (0.10 to 0.48)	0.77 (0.67 to 0.85)	0.17 (−0.03 to 0.36)	0.22 (0.07 to 0.36)
2	0.66 (0.49 to 0.79)	0.27 (0.05 to 0.48)	0.77 (0.64 to 0.85)	0.16 (−0.06 to 0.36)	0.19 (0.01 to 0.35)
3	0.56 (0.30 to 0.75)	0.27 (0.02 to 0.49)	0.79 (0.66 to 0.88)	0.23 (0 to 0.44)	0.12 (−0.10 to 0.31)
4	0.66 (0.47 to 0.79)	0.17 (−0.11 to 0.42)	0.73 (0.58 to 0.84)	0.11 (−0.17 to 0.36)	0.11 (−0.11 to 0.31)
Probandwise concordance rates^b^					
1	0.24	0.09	0.38	0.06	0.07
2	0.25	0.08	0.35	0.05	0.05
3	0.15	0.07	0.36	0.06	0.03
4	0.24	0.04	0.29	0.03	0.03

^a^ Case definition 1 included children who met *DSM*-*5* ARFID criterion A (avoidant/restrictive eating with clinically significant consequences of the eating behavior); case definition 2, children who met *DSM*-*5* ARFID criteria A and C (eating disturbance not attributable to anorexia nervosa, bulimia nervosa, or body image disturbance); case definition 3, children who met *DSM*-*5* ARFID criteria A, C, and partially D (eating disturbance not attributable to a concurrent medical condition or another mental disorder) excluding children with comorbid medical conditions; and case definition 4, children who met *DSM*-*5* ARFID criteria A, C, and partially D excluding children with comorbid autism.

^b^ Probandwise concordance rates were calculated as 2 × number of concordant pairs / ([2 × number of concordant pairs] + number of discordant pairs). They indicate the probability that a cotwin of a proband is also a proband.

## Results

### Identification of the ARFID Phenotype

Of 33 902 included children, 17 151 (50.6%) were male. We identified 801 children (2.4%) who met criteria for case definition 1 (ARFID criterion A) (Figure; Table 1). After excluding children with parent-reported fear of weight gain, 682 children were classified as having the ARFID phenotype (case definition 2), corresponding to a population prevalence of 2.0% (267 of 682 [39.1%] female). Of these, 458 (67.2%) met *DSM*-*5 *ARFID criterion A1 (weight loss or failure to gain weight/grow) and 345 (50.6%) met *DSM*-*5* ARFID criterion A4 (interference with psychosocial functioning; Table 1). Only a small minority met *DSM*-*5 *ARFID criterion A2 (nutritional deficiency; 4 [0.6%]) or *DSM*-*5* ARFID criterion A3 (dependence on supplements/enteral nutrition; 58 [8.5%]). To control for medical conditions that could potentially explain the eating disturbance, we further excluded 130 children who met ARFID criteria A and C (case definition 3; 552 of 33 902 [1.6%]), and to control for the high heritability of autism, we excluded 128 children with autism from case definition 2 (case definition 4; 554 of 33 902 [1.6%]).

### Model Fitting and Heritability of the ARFID Phenotype

According to likelihood ratio tests, model fits did not deteriorate significantly when quantitative sex limitation was dropped (Table 3). In addition, the ADE-s models including quantitative sex limitation were severely underpowered, as indicated by the large 95% CIs for the A and D variance components, which also included zero for all case definitions (Table 4). We therefore fitted nested models of ADE-s models without sex limitation (eTable 3 in Supplement 1). AE-s models showed the best fit for all 4 case definitions (Table 3). Heritability of the ARFID phenotype (case definition 2) was 0.79 (95% CI, 0.70-0.85), with small but statistically significant contribution from nonshared environment (0.21; 95% CI, 0.15-0.30) and sibling contrast effects (−0.10; 95% CI, −0.15 to −0.05; Table 4). Heritability was very similar across all 4 case definitions (point estimate range, 0.77 to 0.79).

**Table 3.** Model Fit Statistics for Models With Quantitative Sex Limitation and Nested Models by Case Definition^a^^,^^b^

Model	−2LL	Parameters	*df*	Comparison model	Change in χ^2^	Change in *df*	*P* value
**Case definition 1**							
Fully saturated	7393.87	15	33897	NA	NA	NA	NA
ADE-s with quantitative sex limitation	7397.21	11	33905	Fully saturated	3.33	8	.91
ADE-s	7401.35	6	33910	ADE-s with quantitative sex limitation	4.15	5	.53
ADE	7405.52	5	33911	ADE-s	4.17	1	.04
AE-s	7401.35	5	33911	ADE-s	0	1	.99
AE	7411.28	4	33912	ADE-s	9.93	2	.007
E	7568.15	3	33913	ADE-s	166.8	3	<.001
**Case definition 2^c^**							
Fully saturated	6500.44	15	33897	NA	NA	NA	NA
ADE-s with quantitative sex limitation	6505.57	11	33905	Fully saturated	5.13	8	.74
ADE-s	6511.50	6	33910	ADE-s with quantitative sex limitation	5.93	5	.31
ADE	6516.20	5	33911	ADE-s	4.70	1	.03
AE-s	6511.57	5	33911	ADE-s	0.07	1	.80
AE	6523.31	4	33912	ADE-s	11.81	2	.003
E	6648.68	3	33913	ADE-s	137.18	3	<.001
**Case definition 3**							
Fully saturated	5494.67	15	33897	NA	NA	NA	NA
ADE-s with quantitative sex limitation	5504.64	11	33905	Fully saturated	9.98	8	.27
ADE-s	5510.4	6	33910	ADE-s with quantitative sex limitation	5.76	5	.33
ADE	5518.7	5	33911	ADE-s	8.30	1	.004
AE-s	5510.4	5	33911	ADE-s	0	1	>.99
AE	5524.35	4	33912	ADE-s	13.95	2	.001
E	5611.58	3	33913	ADE-s	101.18	3	<.001
**Case definition 4**							
Fully saturated	5534.31	15	33897	NA	NA	NA	NA
ADE-s with quantitative sex limitation	5543.94	11	33905	Fully saturated	9.62	8	.29
ADE-s	5547.95	6	33910	ADE-s with quantitative sex limitation	4.01	5	.55
ADE	5552.79	5	33911	ADE-s	4.84	1	.03
AE-s	5548.66	5	33911	ADE-s	0.71	1	.40
AE	5561.53	4	33912	ADE-s	13.58	2	.001
E	5642.51	3	33913	ADE-s	94.56	3	<.001

Abbreviations: −2LL, −2 × log-likelihood; ARFID, avoidant restrictive food intake disorder; NA, not applicable.

^a^ Case definition 1 included children who met *DSM*-*5* ARFID criterion A (avoidant/restrictive eating with clinically significant consequences of the eating behavior); case definition 2, children who met *DSM*-*5* ARFID criteria A and C (eating disturbance not attributable to anorexia nervosa, bulimia nervosa, or body image disturbance); case definition 3, children who met *DSM*-*5* ARFID criteria A, C, and partially D (eating disturbance not attributable to a concurrent medical condition or another mental disorder) excluding children with comorbid medical conditions; and case definition 4, children who met *DSM*-*5* ARFID criteria A, C, and partially D excluding children with comorbid autism.

^b^ A indicates additive genetic variance component, D, dominant genetic variance component, E, nonshared environmental variance component, -s, sibling contrast effects.

^c^ Case definition 2 best reflects children with the ARFID phenotype.

**Table 4.** Variance Component Estimates for Model With Quantitative Sex Limitation and the Final Model Without Sex Limitation^a^^,^^b^

Case definition	Model estimate (95% CI)											
	Model ADE-s with quantitative sex limitation									Final model AE-s without sex limitation		
	Female			Male			-s			A	E	-s
	A	D	E	A	D	E	Same-sex male twins	Same-sex female twins	Opposite-sex twins			
1	0.66 (0-0.81)	0.02 (0-0.72)	0.32 (0.19-0.57)	0.74 (0 to 0.89)	0.09 (0-0.86)	0.17 (0.11-0.28)	−0.10 (−0.17 to 0)	−0.04 (−0.13 to 0.09)	−0.06 (−0.16 to 0.30)	0.78 (0.69 to 0.84)	0.22 (0.16-0.31)	−0.09 (−0.13 to −0.04)
2^c^	0.57 (0.04-0.81)	0.11 (0-0.53)	0.32 (0.19-0.56)	0.83 (0 to 0.90)	0 (0-0.84)	0.16 (0.10-0.26)	−0.13 (−0.19 to −0.04)	−0.01 (−0.11 to 0.10)	−0.09 (−0.19 to 0.28)	0.79 (0.70 to 0.85)	0.21 (0.15-0.30)	−0.10 (−0.15 to −0.05)
3	0.66 (0-0.81)	0 (0-0)	0.34 (0.19-0.70)	0.85 (0.16 to 0.91)	0 (0-0)	0.15 (0.09-0.24)	−0.12 (−0.19 to 0.05)	−0.07 (−0.19 to 0.07)	−0.14 (−0.23 to 0.27)	0.79 (0.70 to 0.86)	0.21 (0.14-0.30)	−0.12 (−0.17 to −0.06)
4	0.32 (0-0.69)	0.36 (0-0.79)	0.32 (0.19-0.58)	0.79 (0 to 0.87)	0.02 (0-0.84)	0.19 (0.11-0.31)	−0.13 (−0.20 to 0.04)	−0.02 (−0.13 to 0.09)	−0.08 (−0.23 to 0.27)	0.77 (0.67 to 0.84)	0.23 (0.16-0.33)	−0.11 (−0.16 to −0.06)

Abbreviation: ARFID, avoidant restrictive food intake disorder.

^a^ Case definition 1 included children who met *DSM*-*5* ARFID criterion A (avoidant/restrictive eating with clinically significant consequences of the eating behavior); case definition 2, children who met *DSM*-*5* ARFID criteria A and C (eating disturbance not attributable to anorexia nervosa, bulimia nervosa, or body image disturbance); case definition 3, children who met *DSM*-*5* ARFID criteria A, C, and partially D (eating disturbance not attributable to a concurrent medical condition or another mental disorder) excluding children with comorbid medical conditions; and case definition 4, children who met *DSM*-*5* ARFID criteria A, C, and partially D excluding children with comorbid autism.

^b^ A indicates additive genetic variance component, D, dominant genetic variance component, E, nonshared environmental variance component, -s, sibling contrast effects.

^c^ Case definition 2 best reflects children with the ARFID phenotype.

## Discussion

In light of the lack of large-scale epidemiological twin data on ARFID, we leveraged existing data to create 4 definitions of an ARFID phenotype. Combining data from parent reports and national health registers, we identified 682 children (2.0%) with the ARFID phenotype and found that the ARFID phenotype is highly heritable. ARFID heritability was 0.79 (95% CI, 0.70-0.85), placing it among the most heritable of psychiatric disorders (autism: 0.64-0.91^36^; schizophrenia: 0.79^37^; ADHD: 0.77-0.88^38^; bipolar disorder: 0.50-0.71^39^). Moreover, the heritability of the ARFID phenotype was higher than that of other eating disorders, namely anorexia nervosa (0.48-0.74), bulimia nervosa (0.55-0.61), and binge-eating disorder (0.39-0.57).^22^ Our results extend and confirm previous twin studies of other feeding-related phenotypes of moderate to high heritability, such as appetite (0.53-0.84),^19^ food fussiness (0.46-0.78),^14,16^ and food neophobia (0.58-0.78).^16,17,18^ In line with other psychiatric phenotypes,^40^ we found the twin-based heritability of ARFID to be higher than the heritability estimated based on common genetic variants.^21^

Excluding individuals with autism and medical conditions that could potentially explain the eating disturbance led to only very minor changes in heritability estimates, suggesting that these conditions did not account for the high heritability. Interestingly, our twin models revealed sibling contrast effects for ARFID, which are commonly observed in neurodevelopmental disorders, including autism and ADHD,^41,42^ suggesting that parents’ ratings of their twins’ eating problems might amplify differences between their twins. Modeling these contrast effects led to an increase in the heritability estimate from 0.67 (in the AE model) to 0.79 (in the AE-s model). Qualitative sex differences (ie, different genetic and environmental influences in male twins vs female twins) did not seem to play an important role, whereas there was some indication for a higher heritability in male twins (ie, quantitative sex difference). Although these were not significant, our models including sex limitations were underpowered, and sex differences need to be tested in future studies with larger sample sizes.

To construct the ARFID phenotype, we were limited to existing data in CATSS, NPR, and PDR. Most cases (760 of 801 [94.9%]) were identified via the parent-reported gate items, “Has he/she ever had a period after age 5 when he/she only wanted to eat particular types of food?” and “Is he/she particularly sensitive to certain flavours, smells, or consistencies?”^43^ (as opposed to being identified with a feeding or eating disorder between age 6 and 12 years; Figure). Therefore, the ARFID phenotype derived in this study is likely to reflect cases that include a sensory-based avoidance component (typically associated with selective eating). This is relevant as genetic and environmental influences might be differentially implicated across predominant ARFID presentations, for instance, the ARFID phenotype in people who had adverse conditioning experiences, such as choking on food, might have a larger environmental contribution. Sensory-based avoidance is the most common presentation (62% to 73%) of ARFID in children,^6,8^ and the presentations are in no way mutually exclusive; more than half of children with ARFID have mixed presentations of sensory-based avoidance with fear-based avoidance or sensory-based avoidance with lack of interest.^6^ Indeed, in line with a large Canadian cohort,^44^ most children with ARFID in the present study were identified via *DSM*-*5* criterion A1 (low weight/failure to thrive; 458 of 682 [67.2%]), which is more commonly associated with lack of interest and fear-based avoidance than with sensory-based avoidance.^45,46^ Future studies aimed at delineating differences in biological and environmental risk factors based on predominant clinical characteristics will require larger samples and more extensive phenotyping.

### Strengths and Limitations

Our study has several strengths and limitations. We optimized existing data resources to provide the first heritability estimates of ARFID based on a sample size larger than typically reported in single-site clinical samples. By triangulating questionnaire and health register data, we accessed many different indicators of ARFID to carefully define the phenotype and specify exclusions for sensitivity analyses. Although it is a limitation that our algorithm-derived definition of ARFID has not been validated by clinical assessments, prevalence and sex distribution were consistent with available published estimates (prevalence, 0.3% to 3.2%^8,47,48^; sex distribution, approximately 1:1, with some studies finding a slight female preponderance^8,47^ and others finding a slight male preponderance^49,50^), providing some confidence in the phenotype. Our study focused on ARFID in children aged 6 to 12 years, yet the disorder is not confined to the childhood years.^51,52^ Subsequent studies using different designs and samples should also include adults to further characterize this illness across the life span. Few cases were identified via *DSM*-*5* ARFID criteria A2 (nutritional deficiency) and A3 (dependence on supplements). The distribution of criteria A1 to A4 is heavily affected by method and setting of ascertainment, and it has previously been shown that criteria A1 and A4 are the most prevalent criteria when screening from the general population,^8,44^ whereas criteria A2 and A3 tend to be more prevalent in clinical samples.^3^ However, that the NPR does not contain diagnoses given in primary health care—a setting in which nutritional deficiencies in children would be likely to be detected and registered—might have additionally contributed to the low prevalence of criteria A2 and A3 in the present study. Finally, even with a sample of approximately 34 000 twins, analyses were underpowered for testing sex differences. Future research should estimate ARFID heritability in even larger samples including older individuals by using validated measures appropriate for epidemiological studies, which are expected to be available in the upcoming years.

## Conclusions

This study shows that, given the similar prevalence figures and sex distribution, existing register-based epidemiological data may be used to approximate ARFID and that the resulting broad ARFID phenotype is highly heritable—with significant contributions from nonshared environmental factors—and distinguishable from other eating disorders characterized by fear of weight gain and older average age of onset. The high heritability of the ARFID phenotype provides strong support for future twin and molecular genetic studies of ARFID.

## Notes

Supplement 1. **eTable 1**. Variables from the Child and Adolescent Twin Study in Sweden (Parent Reports), the National Patient Register (Diagnostic and Procedure Codes), and the Prescribed Drug Register (ATC Codes) Used to Evaluate *DSM*-*5* ARFID Criteria A, C, and D **eTable 2.** Assumption Testing for Models With Qualitative and Quantitative Sex Limitation by Case Definition **eTable 3.** Variance Component Estimates for ADE-s Models Without Sex Limitation and Nested Models by Case Definition (Final Models in Bold)

Supplement 2. Data Sharing Statement
