# Supplementary material for: Etiology of the Broad Avoidant Restrictive Food Intake Disorder Phenotype in Swedish Twins Aged 6 to 12 Years
Source: JAMA Psychiatry. 2023 Feb 1;80(3):260–9. doi: 10.1001/jamapsychiatry.2022.4612 (PMC9978946; doi:10.1001/jamapsychiatry.2022.4612)
Supplement: Supplement 1. — eTable 1. Variables from the Child and Adolescent Twin Study in Sweden (Parent Reports), the National Patient Register (Diagnostic and Procedure Codes), and the Prescribed Drug Register (ATC Codes) Used to Evaluate DSM-5 ARFID Criteria A, C, and D eTable 2. Assumption Testing for Models With Qualitative and Quantitative Sex Limitation by Case Definition eTable 3. Variance Component Estimates for ADE-s Models Without Sex Limitation and Nested Models by Case Definition (Final Models in Bold) [file jamapsychiatry-e224612-s001.pdf]

Supplemental Online Content

Dinkler L, Wronski ML, Lichtenstein P, et al. Etiology of the broad avoidant restrictive food intake disorder phenotype in Swedish twins aged 6 to 12 years. *JAMA Psychiatry*. Published online February 1, 2023.
doi:10.1001/jamapsychiatry.2022.4612

eTable 1. Variables from the Child and Adolescent Twin Study in Sweden (Parent Reports), the National Patient Register (Diagnostic and Procedure Codes), and the Prescribed Drug Register (ATC Codes) Used to Evaluate *DSM-5* ARFID Criteria A, C, and D
eTable 2. Assumption Testing for Models With Qualitative and Quantitative Sex Limitation by Case Definition
eTable 3. Variance Component Estimates for ADE-s Models Without Sex Limitation and Nested Models by Case Definition (Final Models in Bold)

This supplementary material has been provided by the authors to give readers additional information about their work.

© 2023 Dinkler L et al. *JAMA Psychiatry*.

eTable 1. Variables from the Child and Adolescent Twin Study in Sweden (Parent Reports), the National Patient Register (Diagnostic and Procedure Codes), and the Prescribed Drug Register (ATC Codes) Used to Evaluate *DSM-5* ARFID Criteria A, C, and D

**DSM-5 ARFID criterion**	**Name in Figure 1**	**Description of diagnosis, procedure, prescribed medication, or parent-reports**	**Included codes for diagnosis (ICD-9/ICD-10), procedure, prescribed medication (ATC); and response options for parent-reports**	**Exclusions**
A - Feeding or eating disorder dx	Dx Feeding disorders	Feeding disorder of infancy or childhood	F98.2	-----
	Dx Feeding difficulties and mismanagement	Feeding difficulties and mismanagement	783.3, R63.3	-----
	Dx Other/unspecified eating disorder	Other and unspecified disorders of eating	307.5, F50.8	-----
	Dx Anorexia nervosa	Anorexia nervosa	307.1, F50.0, F50.1	-----
	Dx Eating disorder not otherwise specified	Eating disorder not otherwise specified (EDNOS)	F50.9	-----
A - Avoidant/ restrictive eating	(P) Only eats particular types of food > age 5	Has he/she ever had a period after age 5 when he/she only wanted to eat particular types of food?	Yes<br>Yes, to some extent	No
	(P) Very sensitive to flavors/smells/consistencies	Is he/she particularly sensitive to certain flavours, smells, or consistencies?	Yes	Yes, to some extent<br>No
	Dx Loss of appetite	Anorexia (Loss of appetite)	783.0, R63.0	
A1	Dx weight loss/failure to thrive	Other lack of expected normal physiological development in childhood (failure to gain weight/thrive)	783.4, R62.8	-----
		Abnormal weight loss	783.2, R63.4	-----
	Tx weight gain/retaining weight	Weight measurement	AV112	-----
		Assessment of weight maintenance functions	PE006	-----
		Support for weight gain	QE010	-----
	(P) No weight gain/underweight >1 year	Has he/she ever failed to gain enough weight for more than a year or been underweight?	Yes<br>Yes, to some extent	No
	(P) Current BMI <5th percentile	BMI calculated as kg/m2 based on parent-reported height and weight of the twin; BMI < 5th percentile defined by sex and age based on the current sample (male age 9: n=13,858; female age 9: 13,634; male age 12: n=3,293; female age 12: 3,117)	BMI < -2SD	
A2	Dx nutritional anemia	Nutritional anemias	280.1, 280.8, 280.9, 281 D50.1, D50.8, D50.9, D51.3, D51.8, D51.9, D52.0, D52.8, D52.9, D53	D50.0, D51.0, D51.1, D52.2, D52.1 (deficiencies due to blood loss, malabsorption, and intrinsic factors; drug-induced deficiencies)
	Dx nutritional deficiency	Malnutrition & other nutritional deficiencies	260-269, E40-E46, E50-E64	268, E55 (Vitamin D deficiency)

© 2023 Dinkler L et al. *JAMA Psychiatry*.

DSM-5 ARFID criterion	Name in Figure 1	Description of diagnosis, procedure, prescribed medication, or parent-reports	Included codes for diagnosis (ICD-9/ICD-10), procedure, prescribed medication (ATC); and response options for parent-reports	Exclusions
A3	Rx vitamins	Prescribed vitamins	A11	A11CC (Vitamin D)
	Rx minerals	Prescribed minerals	A12	
	Rx for anemias	Prescriptions for anemias	B03	
	Rx parenteral nutrition	Prescribed parenteral nutrition	B05BA	
	Rx infusion concentrates	Prescribed infusion concentrates	B05X	
	Tx tube feeding	Enteral nutrition treatment via tube	DJ010	
		Tube feeding	DV065	
		Nasogastric or nasogastroduodenal tube	TJD00	
		Other tube in ventricle or duodenum	TJD10	
		Changing the gastrostomy catheter	TJD20	
		Jejunal tube via gastrostomy	TJF10	
		Attention to gastrostomy	V55.1, Z43.1	
		Gastrostomy status	V44.1, Z93.1	
	Tx nutritional (incl. counsel./monitoring)	Nutritional value calculation	AV090-AV092	
		Eating training	DJ012	
		Dietary supplements, dietician assessment	DV043	
		Nutritional treatment	DV051-DV056	
		Monitoring of nutritional intake	QE003	
		Feeding	QN022	
		Dietician	XS912	
		Dietary counselling and surveillance	Z71.3	
A4	Psychosocial impairment caused by (P) Underweight	Have peculiarities or problems relating to underweight caused significant impairment in school, among peers or at home?	Yes<br>Yes, to some extent	No
		Do the peculiarities or problems relating to underweight cause him/her significant suffering?	Yes<br>Yes, to some extent	No
	Psychosocial impairment caused by (P) Sensitivity to taste/smell	Have the problems relating to sensitivity to flavours, smells, or consistencies caused significant impairment in school, among peers or at home?	Yes<br>Yes, to some extent	No
		Do the problems relating to sensitivity to flavours, smells, or consistencies cause him/her significant suffering?	Yes<br>Yes, to some extent	No
C - Weight & shape concerns	Weight & shape concerns	Has he/she appeared to be fearful of gaining weight or becoming fat?	Yes<br>Yes, to some extent	No

© 2023 Dinkler L et al. *JAMA Psychiatry*.

DSM-5 ARFID criterion	Name in Figure 1	Description of diagnosis, procedure, prescribed medication, or parent-reports	Included codes for diagnosis (ICD-9/ICD-10), procedure, prescribed medication (ATC); and response options for parent-reports	Exclusions
D - Medical conditions before age 12y[1]	Malignant & in situ neoplasms	Malignant neoplasms	140–209, C00–C97	
		In situ neoplasms	230–234, D00–D09	
	Endocrine & metabolic disorders	Disorders of thyroid gland	240-246, E00-E07	
		Diabetes mellitus	249-250, E10-E14	
		Other disorders of glucose regulation and pancreatic internal secretion	251, E15-E16	
		Disorders of other endocrine glands	249-259, E20-E35	
		Metabolic disorders	270-279, E70-E90	271.3, E73 (lactose intolerance)
	Cerebral palsy + other paralytic syndromes	Cerebral palsy and other paralytic syndromes	342-344, G80–G83	
	Diseases of digestive system - Diseases of esophagus	Oesophagitis	530, K20	
		Gastro-oesophageal reflux disease with oesophagitis	530, K21.0	
		Other diseases of oesophagus	530, K22	
	Diseases of digestive system - Non-infective enteritis & colitis (IBD)	Crohn disease [regional enteritis]	555, K50	
		Ulcerative colitis	556, K51	
	Diseases of digestive system - Other diseases of intestines	Paralytic ileus and intestinal obstruction without hernia	560, K56	
		Neurogenic bowel, not elsewhere classified	564.8, K59.2	
		Perforation of intestine (nontraumatic)	K63.1	
	Diseases of digestive system - Intestinal malabsorption	Intestinal malabsorption	579, K90	579.0, K90.0 (celiac disease)
	Birth injury to central nervous system	Intracranial laceration and haemorrhage due to birth injury	767.0, P10	
		Other birth injuries to central nervous system	767.4, 767.5, 767.7, P11	
	Congenital malformations	Congenital malformations of the nervous system	740-742, Q00-Q07	
		Cleft lip and cleft palate	749, Q35-Q37	
		Other congenital malformations of the digestive system	750-751, Q38-Q45	
		Chromosomal abnormalities, not elsewhere classified	758-759, Q90-Q99	
	Dysphagia	Dysphagia	787.2, R13	
	Intracranial injury	Intracranial injury	851-854, S06	850, S06.0 (concussion)
D - Autism	A-TAC	Autism - Tics, AD/HD and other Comorbidities inventory (A-TAC), 17 items for autism	Theoretical range: 0-17, cut-off value: >=8.5	
	Dx	Pervasive developmental disorders	299A, F84.0, F84.1, F84.5, F84.8, F84.9	

[1] Diagnostic codes for medical conditions that could potentially exclude an ARFID diagnosis were selected based on their presence in the sample, therefore, not *all* medical conditions that could potentially be an exclusion criterion for ARFID are listed here.

© 2023 Dinkler L et al. *JAMA Psychiatry*.

eTable 2. Assumption Testing for Models With Qualitative and Quantitative Sex Limitation by Case Definition

Model	-2LL	Parameters	df	Δχ2	Δdf	p
Case definition 1						
Fully Saturated	7393.9	15	33897	----	----	----
Equal thresholds within same-sex twin pairs	7394.6	11	33901	0.68	4	0.954
Equal thresholds across zygosity for same-sex twin pairs	7398	9	33903	4.11	6	0.662
Case definition 2						
Fully Saturated	6500.4	15	33897	----	----	----
Equal thresholds within same-sex twin pairs	6501.3	11	33901	0.84	4	0.933
Equal thresholds across zygosity for same-sex twin pairs	6506.2	9	33903	5.79	6	0.447
Case definition 3						
Fully Saturated	5494.7	15	33897	----	----	----
Equal thresholds within same-sex twin pairs	5498	11	33901	3.33	4	0.505
Equal thresholds across zygosity for same-sex twin pairs	5507.4	9	33903	12.75	6	0.047
Case definition 4						
Fully Saturated	5534.3	15	33897	----	----	----
Equal thresholds within same-sex twin pairs	5538.7	11	33901	4.42	4	0.352
Equal thresholds across zygosity for same-sex twin pairs	5543.1	9	33903	8.74	6	0.188

Case definition 1 included children who met DSM-5 ARFID criterion A (avoidant restrictive eating with clinically significant consequences of the eating behavior); case definition 2, children who met DSM-5 ARFID criteria A and C (eating disturbance not attributable to anorexia nervosa, bulimia nervosa, or body image disturbance); case definition 3, children who met DSM-5 ARFID criteria A, C, and partially D (eating disturbance not attributable to a concurrent medical condition or another mental disorder) excluding children comorbid medical conditions; and case definition 4, children who met DSM-5 ARFID criteria A, C, and partially D excluding children with comorbid autism.

© 2023 Dinkler L et al. *JAMA Psychiatry*.

eTable 3. Variance Component Estimates for ADE-s Models Without Sex Limitation and Nested Models by Case Definition (Final Models in Bold)

	**Variance component**			
	**A**	**D**	**E**	**s**
Case definition 1				
ADE-s	0.78 (0.06-0.84)	0.00 (0.00-0.68)	0.22 (0.16-0.32)	-0.09 (-0.13-0.00)
ADE	0.20 (0.00-0.60)	0.51 (0.09-0.79)	0.28 (0.21-0.37)	-----
**AE-s**	**0.78 (0.69-0.84)**	**-----**	**0.22 (0.16-0.31)**	**-0.09 (-0.13--0.04)**
AE	0.67 (0.58-0.75)	-----	0.33 (0.25-0.42)	-----
Case definition 2				
ADE-s	0.68 (0.00-0.85)	0.10 (0.00-0.79)	0.22 (0.15-0.32)	-0.09 (-0.15--0.01)
ADE	0.08 (0.00-0.53)	0.64 (0.16-0.80)	0.28 (0.2-0.38)	-----
**AE-s**	**0.79 (0.70-0.85)**	**-----**	**0.21 (0.15-0.30)**	**-0.10 (-0.15--0.05)**
AE	0.67 (0.57-0.75)	-----	0.33 (0.25-0.43)	-----
Case definition 3				
ADE-s	0.79 (0.04-0.86)	0.00 (0.00-0.73)	0.21 (0.14-0.30)	-0.12 (-0.17--0.06)
ADE	0.07 (0.00-0.57)	0.65 (0.11-0.80)	0.29 (0.20-0.40)	-----
**AE-s**	**0.79 (0.70-0.86)**	**-----**	**0.21 (0.14-0.30)**	**-0.12 (-0.17--0.06)**
AE	0.65 (0.53-0.75)	-----	0.35 (0.25-0.47)	-----
Case definition 4				
ADE-s	0.37 (0.00-0.83)	0.39 (0.00-0.81)	0.25 (0.17-0.36)	-0.08 (-0.16--0.01)
ADE	0.00 (0.00-0.03)	0.69 (0.27-0.78)	0.31 (0.22-0.43)	-----
**AE-s**	**0.77 (0.67-0.84)**	**-----**	**0.23 (0.16-0.33)**	**-0.11 (-0.16--0.06)**
AE	0.62 (0.50-0.73)	-----	0.38 (0.27-0.50)	-----

Case definition 1 included children who met DSM-5 ARFID criterion A (avoidant restrictive eating with clinically significant consequences of the eating behavior); case definition 2, children who met DSM-5 ARFID criteria A and C (eating disturbance not attributable to anorexia nervosa, bulimia nervosa, or body image disturbance); case definition 3, children who met DSM-5 ARFID criteria A, C, and partially D (eating disturbance not attributable to a concurrent medical condition or another mental disorder) excluding children comorbid medical conditions; and case definition 4, children who met DSM-5 ARFID criteria A, C, and partially D excluding children with comorbid autism.

© 2023 Dinkler L et al. *JAMA Psychiatry*.
