# Supplementary material for: Etiology of the Broad Avoidant Restrictive Food Intake Disorder Phenotype in Swedish Twins Aged 6 to 12 Years
Source: JAMA Psychiatry. 2023 Feb 1;80(3):260–9. doi: 10.1001/jamapsychiatry.2022.4612 (PMC9978946; doi:10.1001/jamapsychiatry.2022.4612)
Supplement: Supplement 2. — Data Sharing Statement [file jamapsychiatry-e224612-s002.pdf]

Data Sharing Statement

Dinkler. Etiology of the Broad Avoidant Restrictive Food Intake Disorder Phenotype in Swedish Twins Aged 6 to 12 Years. *JAMA Psychiatry*. Published February 01, 2023. doi:10.1001/jamapsychiatry.2022.4612

Data
Data available: No
